# Bacterial Infections, Antimicrobial Resistance and Antibiotic Therapy

**DOI:** 10.3390/life12040468

**Published:** 2022-03-23

**Authors:** Milan Kolář

**Affiliations:** Department of Microbiology, Faculty of Medicine and Dentistry, Palacký University Olomouc, 775 15 Olomouc, Czech Republic; milan.kolar@fnol.cz

Bacterial infections have been, and are very likely to continue to be, among the most serious problems in medicine. One of the main reasons for this is that a significant proportion of these infections are endogenous, and the etiologic agents originate from the human bacterial flora. In this context, it must be emphasized that the bacterial microbiota is absolutely essential for human life; on the other hand, however, it represents a source of bacterial pathogens potentially implicated in the development of a wide range of infections [1,2]. An integral part of the treatment of bacterial infections is the administration of antibiotics that target etiologic agents. Antibacterial drugs as we know them in today′s medicine have been used for 80 years. Despite the great expansion of antibacterials in the 1960s and 1970s, as documented by the development of a range of new products and their introduction into practice, bacterial infections remain a major issue of increasing importance. Modern medicine is even confronted with the real threat that antibiotics may lose their effect on bacteria and the associated ability to treat bacterial infections. The increasing resistance of bacterial pathogens to antibacterial drugs; for example, the rising prevalence of bacteria producing broad-spectrum beta-lactamases, including metallo-beta-lactamases and carbapenemases, raises the possibility of a return to a new “antibiotic-free era” in which adequate antibiotics will not be available for the treatment of bacterial infections with an etiological role of multidrug-resistant bacteria [3,4].

Antimicrobial resistance (AMR) is an inherent part of the issue of bacterial infections and represents a significant public health problem. The use of antibacterial agents is essential in the treatment of patients with bacterial infections. However, the effectiveness of antibiotics is increasingly limited by the growing resistance of pathogenic bacteria, which significantly increases the likelihood of failure of antibiotic therapy. It can be said that AMR is not only a medical issue but that it affects the entire society as it begins to limit the further development of diagnostic and therapeutic practices in clinical medicine by reducing their success rate in the context of the increased morbidity and mortality of infectious complications due to multidrug-resistant bacteria. Tumbarello et al., reported a mortality rate of as much as 60% in patients with bloodstream infections caused by ESBL-positive enterobacteria following inadequate antibiotic treatment. In contrast, adequate antibiotic therapy was associated with a significantly lower mortality of 19% [5]. In patients with hematologic malignancies and complicating bloodstream infections, Trecarichi et al., documented a 40% mortality rate in the case of multidrug-resistant strains of *Pseudomonas aeruginosa*, as compared with 9% for susceptible strains [6]. By using hemato-oncology patients as an example, it can be demonstrated that the effective initial antibiotic treatment of febrile neutropenia reduces mortality to 2 to 10%. At present, however, there is a considerable danger that due to increasing AMR, the rates will rise. In patients with hematologic malignancies, mortality associated with sepsis caused by ESBL-positive enterobacteria may be 25% or, in the case of positive carbapenemase production, even 69% higher [7,8]. Herkel et al., reported a statistically significant difference in mortality between patients receiving adequate and inadequate antibiotic treatment for ventilator-associated pneumonia. While the former showed 27% mortality, inadequate antibiotic therapy was associated with a rate of 45%, with bacterial pathogens being resistant to initial antibiotics [9]. Rather alarming is the fact that multidrug-resistant bacteria have also been confirmed as part of the normal microbiota. For example, Arnan et al., found ESBL-positive *Escherichia coli* strains in 29% of patients with neutropenia, compared to only 14% at the time of hospital admission [10]. A study conducted in the University Hospital Olomouc, Czech Republic, showed 21% prevalence of ESBL- and AmpC-positive enterobacteria in the gastrointestinal tract of hemato-oncology patients [11]. At the same time, it should be emphasized that genetic analysis revealed identical bacterial agents isolated from the urine and blood and from the gastrointestinal tract in two patients with clinically manifested bacterial infection (urinary tract and bloodstream infection). This suggests that multidrug-resistant bacteria present in the intestinal flora caused serious illness [11]. It should also be reminded that multidrug-resistant bacteria are found in the environment, as confirmed, for example, by studies published in this Special Issue of *Life* [12,13].

The urgency of AMR increases with the requirement for adequate treatment of serious bacterial infections, especially in intensive care patients. In these cases, it is necessary to administer antibacterials as soon as possible, preferably within hours (depending on the diagnosis) [14,15]. However, it is not possible to accurately determine the etiologic agent and its susceptibility to antibiotics over such a short period of time. On the other hand, adequate antibiotic treatment can substantially contribute to a positive therapeutic effect in a particular patient. The problem of AMR is clearly multifactorial and must be addressed by an interdisciplinary approach involving many medical specialties. The key prerequisite for its successful solution is close multidisciplinary cooperation and the implementation of bacterial resistance surveillance, an essential part of which must be the determination of the selection of multidrug-resistant bacteria and the pathways and mechanisms of their spread, including their genetic basis as described, for example, in the study by Bogdanova et al., in this Special Issue [16]. These data are indispensable for establishing the basic principles of a rational antibiotic policy and adequate hygiene and epidemiological measures. This clearly suggests the need for the practical implementation of antibiotic stewardship, a set of measures leading to rational antibiotic treatment based on appropriate selection of antibacterial drugs, the duration of their administration, as well as the route of administration [17,18,19,20]. Such an approach is very comprehensive and includes a range of different activities, in particular:

- Adequate identification of bacterial pathogens and correct interpretation of microbiological results; the aim is to treat infections, not bacterial contamination or colonization;
- Assessing the frequency of bacterial pathogens in individual infections or infectious complications;
- Analysis of bacterial resistance to antibiotics (including sources and routes of spread of multidrug-resistant bacteria) in human, animal, and environmental settings using modern molecular genetic methods;
- Development of local and national guidelines for initial antibiotic treatment;
- Adequate antibiotic prophylaxis.

The overall solution should also involve strict adherence to hygiene and epidemiological measures, the development of new antibacterial drugs and providing the professional and lay public with information on increasing resistance of bacterial pathogens to antibiotic treatment and the decreasing number of suitable medications.

The rational use of antibiotics to treat bacterial diseases is a fundamental requirement of today′s medicine. It must be stressed though that, in many cases, it is not possible to administer antibiotics based on identification of the etiological bacterium and determination of its susceptibility to antibacterial drugs. This is mainly true for acute bacterial infections, where there is a risk of delays. In these cases, it is necessary to use initial (non-targeted) antibiotic therapy. However, this does not mean the administration of antibiotics without consideration; strict adherence to a number of basic principles is needed, which can be defined as follows:

- Knowledge of the normal human microflora;
- Knowledge of the microbes most commonly involved in affecting a particular organ or tissue;
- Information on the epidemiological and possibly nosocomial situation in a particular epidemiological unit;
- Information on antimicrobial resistance of the most common and important bacterial pathogens;
- Knowledge of the microbiological and pharmacological properties of the antibiotics under consideration (spectrum of action, type of action, bioavailability, penetration into tissues, etc.);
- The application of pharmacokinetic/pharmacodynamic parameters and a personalized approach to the patient:
  − Age;
  − Kidney and liver function;
  − Predisposition to allergies;
  − Length of stay in the ward in the case of inpatients;
  − Site of confirmed or suspected bacterial infection;
  − Community-acquired or nosocomial infection;
  − Severity of infection (mild, moderate or severe);
  − Immune system status;
  − Previous antibiotic treatment;
  − Current medication with regard to possible interactions;
  − Results of bacteriological monitoring of the patient.

But what is crucial is the indication for antibiotic therapy. The use of antibiotics is a risk factor in terms of the selection of bacterial strains with a higher degree of primary resistance (e.g., strains of *Pseudomonas aeruginosa*, *Acinetobacter baumannii*, *Stenotrophomonas maltophilia*, or *Burkholderia cepacia* complex), as well as bacteria with secondary, or acquired, resistance (e.g., methicillin-resistant strains of staphylococci, enterobacteria producing broad-spectrum beta-lactamases, or carbapenem-resistant strains of *Pseudomonas aeruginosa*). Therefore, antibiotic treatment should be limited to clinically confirmed or very likely bacterial infections. Administering antibacterials only to “cover the patient” should be rejected as unwarranted and risky. Despite the fact that the use of antibiotics is conditioned by local sources of information, it is necessary to mention other no less important sources of data, namely, the generally valid data on the pathogenesis of bacterial infections and properties of microorganisms, as well as the facts on the properties of antibiotics (pharmacokinetics, excretion, penetration into tissues, etc.), and, last but not least, the results of bacterial resistance surveillance at both national and international levels, such as the EARS-Net database [21].

A modest contribution to addressing AMR, bacterial infections and antibiotic treatment is this Special Issue of *Life*. The authors believe that the published results provide new evidence and ideas to preserve the effect of antibiotics and the ability to treat bacterial infections for the future.

Supported by the Ministry of Health of the Czech Republic project no. AZV NU22-B-112.
